# Transcription Factor β-Catenin Plays a Key Role in Fluid Flow Shear Stress-Mediated Glomerular Injury in Solitary Kidney

**DOI:** 10.3390/cells10051253

**Published:** 2021-05-19

**Authors:** Tarak Srivastava, Daniel P. Heruth, R. Scott Duncan, Mohammad H. Rezaiekhaligh, Robert E. Garola, Lakshmi Priya, Jianping Zhou, Varun C. Boinpelly, Jan Novak, Mohammed Farhan Ali, Trupti Joshi, Uri S. Alon, Yuexu Jiang, Ellen T. McCarthy, Virginia J. Savin, Ram Sharma, Mark L. Johnson, Mukut Sharma

**Affiliations:** 1 Section of Nephrology, Children’s Mercy Hospital and University of Missouri at Kansas City, Kansas City, MO 64108, USA; mhrezaiekhaligh@cmh.edu (M.H.R.); lp935@mail.umkc.edu (L.P.); fali@cmh.edu (M.F.A.); ualon@cmh.edu (U.S.A.); 2 Midwest Veterans’ Biomedical Research Foundation (MVBRF), Kansas City, MO 64128, USA; jianping.zhou@va.gov (J.Z.); varun.boinpelly@va.gov (V.C.B.); mukut.sharma@va.gov (M.S.); 3 Department of Oral and Craniofacial Sciences, School of Dentistry, University of Missouri at Kansas City, Kansas City, MO 64108, USA; johnsonmark@umkc.edu; 4 Children’s Mercy Research Institute, Children’s Mercy Hospital and University of Missouri at Kansas City, Kansas City, MO 64108, USA; dpheruth@cmh.edu; 5 School of Biological Sciences, University of Missouri at Kansas City, Kansas City, MO 64108, USA; duncanrs@umkc.edu; 6 Department of Pathology and Laboratory Medicine, Children’s Mercy Hospital and University of Missouri at Kansas City, Kansas City, MO 64108, USA; regarola@cmh.edu; 7 Kansas City VA Medical Center, Kansas City, MO 64128, USA; vjsavin@gmail.com (V.J.S.); ram.sharma2@va.gov (R.S.); 8 Department of Microbiology, University of Alabama at Birmingham, Birmingham, AL 35487, USA; jannovak@uab.edu; 9 Department of Health Management and Informatics, University of Missouri, Columbia, MO 65211, USA; joshitr@health.missouri.edu; 10 Department of Electrical Engineering and Computer Science, University of Missouri, Columbia, MO 65211, USA; yjm85@mail.missouri.edu; 11 Christopher S. Bond Life Sciences Center, University of Missouri, Columbia, MO 65211, USA; 12 MU Data Science and Informatics Institute, University of Missouri, Columbia, MO 65211, USA; 13 Department of Internal Medicine, The Jared Grantham Kidney Institute, University of Kansas Medical Center, Kansas City, KS 66160, USA; emccarthy@kumc.edu

**Keywords:** podocytes, fluid flow shear stress, glomerular hemodynamics, hyperfiltration, glomerular filtration barrier

## Abstract

Increased fluid flow shear stress (FFSS) in solitary kidney alters podocyte function *in vivo*. FFSS-treated cultured podocytes show upregulated AKT-GSK3β-β-catenin signaling. The present study was undertaken to confirm (i) the activation of β-catenin signaling in podocytes in vivo using unilaterally nephrectomized (UNX) TOPGAL mice with the β-galactosidase reporter gene for β-catenin activation, (ii) β-catenin translocation in FFSS-treated mouse podocytes, and (iii) β-catenin signaling using publicly available data from UNX mice. The UNX of TOPGAL mice resulted in glomerular hypertrophy and increased the mesangial matrix consistent with hemodynamic adaptation. Uninephrectomized TOPGAL mice showed an increased β-galactosidase expression at 4 weeks but not at 12 weeks, as assessed using immunofluorescence microscopy (*p* < 0.001 at 4 weeks; *p* = 0.16 at 12 weeks) and X-gal staining (*p* = 0.008 at 4 weeks; *p* = 0.65 at 12 weeks). Immunofluorescence microscopy showed a significant increase in phospho-β-catenin (Ser552, *p* = 0.005) at 4 weeks but not at 12 weeks (*p* = 0.935) following UNX, and the levels of phospho-β-catenin (Ser675) did not change. In vitro FFSS caused a sustained increase in the nuclear translocation of phospho-β-catenin (Ser552) but not phospho-β-catenin (Ser675) in podocytes. The bioinformatic analysis of the GEO dataset, #GSE53996, also identified β-catenin as a key upstream regulator. We conclude that transcription factor β-catenin mediates FFSS-induced podocyte (glomerular) injury in solitary kidney.

## 1. Introduction

Persistent hyperfiltration is one of the major causes of a loss of glomerular function and the progression of chronic kidney disease (CKD), but the underlying mechanism is not well understood. Hemodynamic parameters, including renal blood flow, glomerular capillary pressure (P_GC_), single nephron glomerular filtration rate (SNGFR), filtration fraction, and decreased hydraulic conductivity associated with glomerular hypertrophy, have been used to study hyperfiltration, leading to a detailed analysis of these parameters [1,2]. Our laboratory and a few other groups have recently started investigating biomechanical forces that mediate the effects of hyperfiltration in the glomerulus [3,4,5,6]. 

Two types of biomechanical forces are believed to mediate the effects of hyperfiltration on glomerular function and structure, namely, tensile stress and fluid flow shear stress (FFSS), which were recently reviewed by Srivastava et al. [5,6]. Podocytes localized in Bowman’s space are most vulnerable to increased biomechanical forces [7,8,9]. Primary processes from the large soma of podocytes further branch into foot processes covering the glomerular basement membrane around the capillary and interdigitate to form slit pore junctions. The increased intra-capillary pressure, working outwardly perpendicular to the direction of blood flow in the capillary, stretches foot processes and generates tensile stress on the basolateral aspect of podocytes [5]. The second biomechanical force, FFSS, is generated on podocyte soma and primary processes by the glomerular ultrafiltrate flowing through Bowman’s space [5,7]. Thus, P_GC_ and SNGFR become the principal determinants of tensile stress and FFSS, respectively [5,6]. We have shown a 1.5–2.0-fold increase in the calculated FFSS over podocytes in solitary kidney in mice and rats [9].

The in vitro application of FFSS to podocytes resulted in an altered actin cytoskeleton, increased the secretion of prostaglandin E_2_ (PGE_2_), and increased the expression of cyclooxygenase (COX)2 and PGE_2_ receptor EP2, but not EP4 [10,11,12]. Additionally, we demonstrated an upregulated COX2-PGE_2_-EP2 axis with an unchanged EP4 expression in unilaterally nephrectomized (UNX) mice, as well as an elevated urinary PGE_2_ in children born with solitary kidney [10,13]. These findings established the significance of the COX2-PGE_2_-EP2 axis in mediating the mechanoreception of FFSS by podocytes.

**Figure 1:**
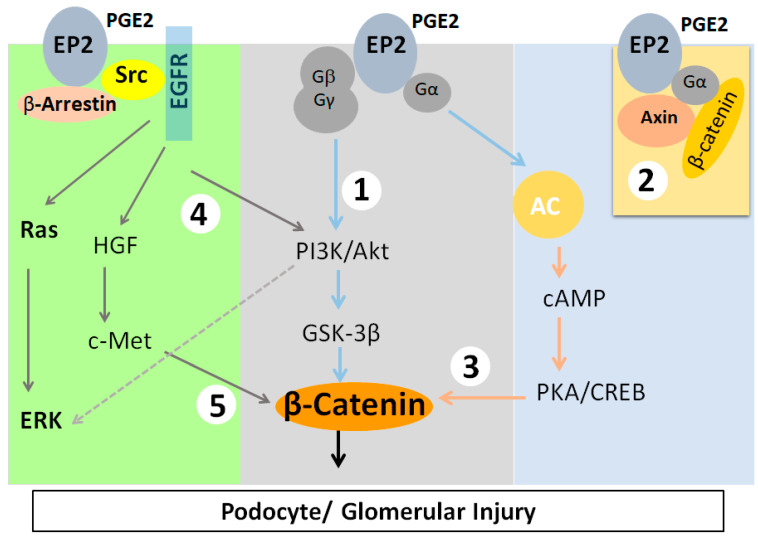
Phosphorylation of β-catenin at Ser552 or Ser675 results in its translocation to the nucleus. EP2 initiates the activation of β-catenin through five known intermediates. As EP2 is a G-protein coupled receptor, it activates the heterotrimeric (αβγ) Gs protein, leading to (**1**) the release of the Gβγ complex, thus activating PI3K/AKT, which phosphorylates and inactivates GSK3β, resulting in the nuclear translocation of active β-catenin (**Middle**), (**2**) binding of the Gα complex to Axin and release of β-catenin from the Axin-β-catenin-GSK3β complex (**Right**), and (**3**) generation of cAMP and activation of PKA, which activate β-catenin. (**4**) The PGE_2_ receptor EP2 recruits β-arrestin 1 and phosphorylates Src-kinase, which transactivates the EGFR signaling network of the PI3K/AKT, HGF/c-Met, and Ras/ERK pathways. (**5**) The transactivation of c-Met by PGE_2_ through EGFR also leads to an increase in nuclear β-catenin (**Left**). EP2 activation and PI3K/AKT also activate extracellular signal-regulated kinase (ERK).

PGE_2_ binding with EP2 receptor results in signaling events that induce β-catenin translocation to the nucleus (Figure 1). Bioinformatics analysis to address the mechanotransduction that converts mechanical stimuli into biochemical changes identified Glycogen Synthase Kinase-3β (GSK3β) using Kinase enrichment analysis and Protein Kinase B (AKT1), GSK3β, and β-catenin through protein–protein interaction network analysis. Validation of these analyses using immortalized mouse podocytes led to the conclusion that AKT-GSK3β-β-catenin signaling plays a key role in podocyte response to FFSS in vitro [14,15]. 

Intracellular levels of β-catenin, a transcription factor, are normally kept low by the association of phosphorylated β-catenin with a degradation complex that targets it to the ubiquitin-proteasome degradation pathway in the absence of Wnt ligand. In the presence of Wnt bound to membrane receptor complex (LRP5/6 and frizzled), it is released from the degradation complex for translocation to the nucleus. Phosphorylation by Casein Kinase at Ser45, followed by GSK3β, at Ser33, Ser37, and Thr41 directs β-catenin for degradation, whereas phosphorylation by AKT at Ser552 and by Protein Kinase A (PKA) at Ser675 results in the nuclear translocation of β-catenin [16,17,18]. Activated β-catenin signaling has been identified in Adriamycin-induced podocyte injury and albuminuria, oxidative stress-induced podocyte dysfunction, diabetic nephropathy, and focal segmental glomerulosclerosis [19,20]. The significance of β-catenin in solitary kidney injury and dysfunction is not known. 

The present studies were undertaken to assess and confirm the upregulation of AKT-GSK3β-β-catenin signaling using a mouse model of UNX. We performed UNX in TOPGAL mice, a specific reporter mouse line with β-galactosidase activity as a direct in vivo readout for activated β-catenin signaling [21]. The increased FFSS in solitary kidney following UNX resulted in the activation of β-catenin in TOPGAL mice. Next, we carried out in vitro experiments using cultured podocytes, which demonstrated phosphorylation of β-catenin at Ser552 as the main driver of β-catenin nuclear translocation. Additionally, we analyzed the publicly available datasets in the gene expression omnibus (GEO) database repository, which showed an upregulation of AKT-GSK3β-β-catenin in solitary kidney following UNX in mice [22,23]. Thus, studies using in vivo UNX TOPGAL mice, cultured podocytes in vitro, and bioinformatic data analysis on UNX mice provide combined evidence supporting our original in vitro findings and a better understanding of the molecular events associated with hyperfiltration-induced glomerular injury in solitary kidney. 

## 2. Materials and Methods

### 2.1. Unilateral Nephrectomy of TOPGAL Mice 

Animal studies involving TOPGAL mice were carried out using protocols approved by the Institutional Animal Care and Use Committee (IACUC), Institutional Biosafety Committee/Subcommittee on Research Safety (IBC/SRS), and the Research and Development (R&D) Committee at the VA Medical Center, Kansas City, MO. The study was carried out in compliance with the ARRIVE guidelines. All methods were performed in accordance with the relevant guidelines and regulations. The mice were maintained at AAALAC-approved facilities with unrestricted access to food and water under light/dark cycles of 12/12 h. The TOPGAL [Tg(TCF/Lef1-lacZ)34Efu] mouse carries a lacZ transgene on a CD1 background (Stock #4623, The Jackson Laboratory, Bar Harbor, ME, USA). The transgene contains the lacZ gene under the control of a promoter consisting of three consensus lymphoid enhancer binding factor 1/transcription factor 3 (LEF/TCF)-binding motifs upstream of a minimal Fos promoter [21]. This allele is responsive to canonical Wnt/β-catenin signal transduction. Thus, the TOPGAL mouse is a specific reporter strain, with β–galactosidase activity as a direct in vivo readout for activated β–catenin signaling. 

Four-week-old TOPGAL mice (*n* = 47) underwent unilateral nephrectomy (UNX) to remove the right kidney (UNX) or sham operation (Sham). The animals recovered in their cages following the surgery, and the left kidney was harvested for analysis at 4 or 12 weeks following UNX. The day prior to the harvesting of the kidneys, urine was collected for urine albumin and creatinine measurements. At the time of the tissue retrieval, an intracardiac perfusion was performed with ice cold 4%PFA in PBS (pH 7.8) for 10 min at 4.4 mL/min, followed by ice cold 18% sucrose in PBS (pH 7.8) for 10 min at 4.4 mL/min. The fixative solutions were prepared and filtered to remove small particles which otherwise may block the capillaries. The resected kidney was then incubated overnight in 30% sucrose in PBS (pH 7.8) on ice [24]. A part of the kidney was embedded in optimal cutting temperature compound (OCT) and flash frozen in liquid nitrogen. A part of the kidney was fixed in 10% formalin, processed, and embedded in paraffin.

### 2.2. Urine Albumin and CREATININE 

Urine albumin was measured in triplicates using an Albuwell M kit (#1011, Exocell Inc., Philadelphia, PA, USA), following the manufacturer’s instructions. Urine creatinine was measured in duplicates using a companion creatinine kit (#1012, Exocell Inc., Philadelphia, PA, USA), following the manufacturer’s instructions. 

### 2.3. Morphometric Analysis of Glomeruli in the TOPGAL Mouse Kidney

Paraffin-embedded tissue was sectioned at 3–5 μm and stained with Periodic Acid-Schiff (PAS) stain. PAS-stained kidney sections were evaluated for glomerular characteristics, including the size, mesangial matrix, and mesangial cell count. The glomerular area and perimeter included only the glomerular tufts and not the Bowman’s space or the parietal epithelial cells. The mesangial area included the PAS-stained matrix and cells. The mesangial cell counts were derived from the most proliferative mesangial stalk of each glomerulus, but not immediately adjacent to a vascular stalk. The counts were taken from cross sections and not the longitudinal section of the stalk, as described by Roberts et al. [25]. Images were obtained using an Olympus BX60 (Hamburg, Germany) for light microscopy and analyzed using the Image J software suite (National Institute of Health and the Laboratory for Optical and Computational Instrumentation at the University of Wisconsin, Madison, WI, USA) [26].

### 2.4. X-Gal Staining for β-Galactosidase Expression in TOPGAL Mice

The tissue sections (8–10 μm in thickness) were fixed by immersion in 2% glutaraldehyde, 0.01% sodium deoxycholate, 0.02% IGEPAL-CA630, and 1 mM MgCl_2_ in PBS [pH = 7.8]) for 10 min and washed in a LacZ wash buffer (2 mM MgCl_2_, 0.01% Na-deoxycholate, and 0.02% NP40 in PBS). The sections were immersed and incubated overnight with gentle rocking in a staining solution (1 mg/mL X-gal, 5 mM potassium ferricyanide, 5 mM potassium ferrocyanide, and 2 mM MgCl_2_ in PBS, pH = 8.0) at 30 °C in the dark for 24 h in a humidified atmosphere [24]. The tissue sections were washed with PBS, counterstained using a nuclear fast red staining solution, and washed with water for 1–2 min. Finally, the tissue sections were dehydrated through grades of ethanol and xylene and mounted in a non-aqueous mounting fluid. Using the images obtained after X-gal staining, endothelial cells were localized on the intraluminal aspect of the glomerular capillary, while podocytes were on the extraluminal aspect of the capillary wall. Semi-quantitative analysis was performed using a scale of 0–4 for X-gal staining within the glomerulus. Each glomerulus examined was assigned a score based upon the percentage of total podocytes that showed positive staining: 0 (no staining), 1 (<10% of podocytes), 2 (10–25% of podocytes), 3 (25–50% of podocytes), or 4 (>50% of podocytes staining).

### 2.5. Immunofluorescence Staining of TOPGAL Mouse Kidneys and Immortalized Mouse Podocytes

The tissue sections (4 μm) were fixed at 4 °C in 1:1 acetone and alcohol for 10–15 min, washed with PBS, and blocked in 5% donkey serum for 3 h at room temperature. The blocked sections were washed with PBS and incubated with primary antibodies for Podocalyxin (Goat Polyclonal Antibody from R&D, Catalog number AF1556, 1:1000 dilution), Total β-catenin (Rabbit Monoclonal Antibody from Cell Signaling, cs-8480S, 1:100 dilution), phospho-β-catenin (Ser675, Rabbit Monoclonal Antibody from Cell Signaling, cs-4176S, 1:100), phospho-β-catenin (Ser552, Rabbit Monoclonal Antibody from Cell Signaling, cs-5651S, 1:200 dilution), or β-galactosidase (Rabbit Polyclonal Antibody from Sigma, Catalog Number A-11132, 1:100 dilution) in a blocking solution overnight at 4 °C. The tissue slices were then washed and incubated with fluorescent-tagged secondary antibody (Donkey anti-rabbit Alexa Fluor 488, Invitrogen Catalog # A-21206 and Donkey anti-goat Alexa Fluor 594, Invitrogen Catalog # A-32758) for 1 h at room temperature and washed. The tissue sections were counterstained with Evan’s Blue for 4 min, washed, and mounted in an aqueous mounting fluid. 

The cultured podocytes were fixed in 4% paraformaldehyde in PBS for 15 min at room temperature, washed in PBS, permeabilized in Triton X-100 0.1% in PBS for 10 min, and washed again. The cells were blocked in 1% goat serum/2.5% BSA in PBS for 2–3 h at room temperature, washed, and then incubated with primary antibodies in a blocking solution overnight at 4 °C, as described above for the tissue sections. The cells were washed and incubated in Alexa Fluor 488-tagged goat anti-rabbit secondary antibody (1:200 dilution) for 1 h at room temperature, washed, and mounted in an aqueous mounting fluid.

### 2.6. Confocal Microscopy and Measurement of Immunofluorescence Intensity

Confocal microscopy with Z-stacking was performed on a Zeiss LSM 510 META microscope with an LSM 510 laser module. We obtained images at 63× magnification (in immersion oil). The z-stack depth of the confocal images is approximately 4 μm (+/− 1.5 μm) with a an optical section (z-slice) depth of 0.317 μm. We converted stacked images into maximum-intensity-projection images for analysis. The cells were treated with FFSS in triplicate, and 3–5 images were obtained per experiment. The photomultiplier tube voltage was kept constant throughout image acquisition to ensure comparable relative fluorescence intensities for all experimental conditions. Quantitative analysis of confocal microscope images was carried using the open-source Image-J/FIJI software to determine the predominant subcellular location of different phospho-β-catenin [27]. The fluorescent staining for β-catenin protein in the nucleus was quantitated by measuring the relative fluorescence units (RFU) selected in the region of interest. We measured the net fluorescence integrated density in the nucleus using the area of the nucleus and the mean intensity of β-catenin immunofluorescence within the nucleus. We measured a minimum of 50 nuclei at each experimental time point. A similar protocol was followed for quantification in kidney tissue from TOPGAL animals. 

### 2.7. Podocyte Cell Culture 

Conditionally immortalized mouse podocytes containing thermosensitive tsA58 mutant T-antigen (kindly provided by Dr. Peter Mundel) were seeded on collagen-I coated 75 cm^2^ polystyrene flasks and first propagated in RPMI 1640 containing L-glutamine, 10% fetal bovine serum, 100 units/mL penicillin, and 0.1 mg/mL streptomycin (Invitrogen, Carlsbad, CA, USA) supplemented with 10 units/mL of γ-interferon (Cell Sciences, Norwood, MA, USA) under permissive conditions at 33 °C, with 95% humidity and 5% CO_2_ [28]. The cells were then transferred to non-permissive conditions (37 °C without γ-interferon) to induce differentiation. The cells on standard glass slides (3 slides/dish, 12 mL medium) were maintained for differentiation and studied on day 14.

### 2.8. Fluid Flow Shear Stress (FFSS) Application 

Fluid flow shear stress was applied to the differentiated podocytes using a FlexCell Streamer Gold apparatus (FlexCell International, Hillsborough, NC, USA), as described previously [10,14]. FFSS was applied at 2 dynes/cm^2^ for 2 h and untreated podocytes (control group) were placed in the same incubator, without exposure to FFSS. The slides were returned to the dishes for recovery up to 24 h at 37 °C under a 5% CO_2_-humidified atmosphere. The samples obtained from untreated podocytes at the end of the FFSS treatment, and at 2 h and 24 h following the FFSS treatment, were termed Control, End-FFSS, Post-2 h, and Post-24 h, respectively.

### 2.9. Gene Expression Omnibus Dataset on Unilaterally Nephrectomized Mice

The Gene Expression Omnibus (GEO, NCBI, (http://www.ncbi.nlm.nih.gov/geo, access on 24 July 2017) public functional genomics data repository was searched for “uninephrectomy” and filtered for “*Mus musculus*”, which returned 58 hits (as of 24 July 2017). We excluded results for uninephrectomy associated with ischemia-perfusion injury, diabetic nephropathy, drug treatment, and pooled samples. We identified the GEO dataset, GSE 53996, titled “Effects of high-fat induced obesity on gene expression in mouse kidney”. In the animal protocol used for obtaining this GEO dataset, male C57/BJ mice aged 6 weeks were randomly assigned to left uninephrectomy (UNX) or sham procedures and fed a high-fat diet or control chow diet. The mice were divided into 4 groups: Sham-chow, UNX-chow, Sham-HFD, and UNX-HFD. All mice were sacrificed under anesthesia at 20 weeks after surgery, and their kidneys were harvested. Microarray was performed on Agilent-026655 Mouse GE 4x44K v2 [29,30]. To evaluate the transcriptomic changes induced by unilateral nephrectomy alone, avoiding the effect of the high-fat diet, the present bioinformatics analysis included only the groups maintained on normal chow. These included the sham samples (*n* = 4): GSM1305179 LFD-sham_1, GSM1305180 LFD-sham_2, GSM1305181 LFD-sham_3, GSM1305182 LFD-sham_4, and UNX samples (*n* = 4): GSM1305183 LFD-UNX_1, GSM1305184 LFD-UNX_2, GSM1305185 LFD-UNX_3, and GSM1305186 LFD-UNX_4. 

### 2.10. Functional Annotation, Pathway, and Network Analyses Using Ingenuity Pathway Analysis (IPA) 

To identify over-represented canonical pathways, networks, and upstream regulators, differentially expressed genes (*p* < 0.05) between sham and unilateral nephrectomy were submitted for the Ingenuity Pathways Knowledge Base tool (IPA, Ingenuity Systems, Inc., Redwood City, CA, USA). Core analysis was performed by comparing the significant genes with the IPA knowledge base, comprising curated pathways within the program on 24 July 2017. IPA predicted significant biological functions and pathways (*p* < 0.05, Fischer’s exact test) affected in the kidney following unilateral nephrectomy. In addition, IPA was used to predict the upstream biological regulators and the downstream effects on cellular and organismal biology. In the analysis of the upstream regulators, such as transcription factors, kinases, receptors, etc., the further the activation z-score is away from zero, the more likely it is that the direction of change of the target genes is consistent with the upstream regulator being in an activated or inhibited state. The settings for the core analyses were as follows: Ingenuity Knowledge Base; Endogenous Chemicals included Direct and Indirect relationships; molecules per pathway: 35; and networks per analysis: 25. 

### 2.11. Statistics

We used the SPSS 23 statistical software for the preparation of the statistics and graphs. ANOVA was used for three-group comparisons of the podocyte experiments, and Students’ *t*-test was used for two-group comparisons. A *p*-value < 0.05 was considered significant.

## 3. Results

Previously, we demonstrated that FFSS induces the activation of the “PGE_2_-COX2-EP2” axis and the “AKT-GSK3β-β-catenin” pathway in podocytes in vitro. We validated the role of the COX2 and EP2 in vivo model of UNX [10,11,12,14]. The present study was undertaken to determine if in vivo FFSS following UNX in mice would support the original in vitro findings concerning the role of β-catenin. To this end, we used TOPGAL mice and immortalized mouse podocytes to demonstrate the activation and nuclear translocation of β-catenin. We also applied bioinformatic analysis to determine if publicly available data from UNX mice support our in vivo and in vitro findings. 

### 3.1. Unilateral Nephrectomy Affected Growth, Glomerular Morphology, and β-Catenin in TOPGAL Mice

**Figure 2:**
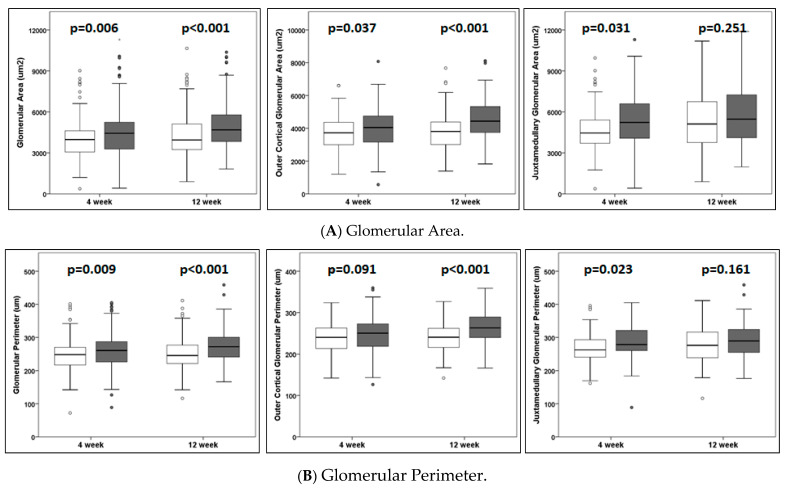

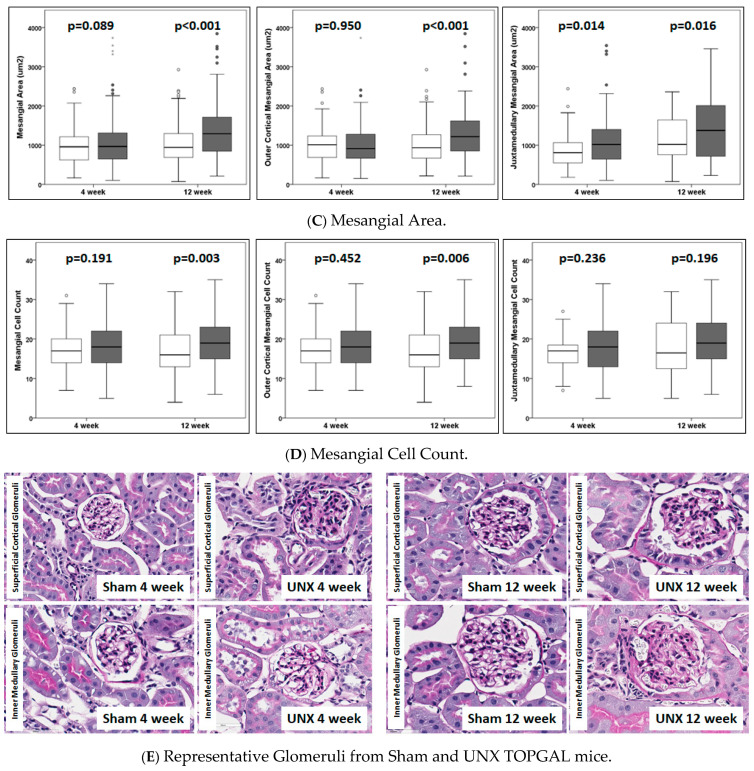
Box-plot distribution of (**A**) the glomerular area, (**B**) glomerular perimeter, (**C**) mesangial area, (**D**) mesangial cell count, and (**E**) representative glomeruli from superficial cortical glomeruli (upper row) and deep intramedullary glomeruli (lower row) in TOPGAL animals at 4 and 12 weeks following unilateral nephrectomy or sham treatment at 4 weeks of age. These measurements were performed on 15 glomeruli (10 outer cortical glomeruli and 5 inner juxtamedullary glomeruli) in kidneys stained with Periodic Acid-Schiff stain at 4 weeks and 12 weeks. The data from all glomeruli are shown in the left panel, outer cortical glomeruli in the middle panel, and inner juxtamedullary glomeruli in the right panel. There were 12 sham and 12 UNX animals at 4 weeks and 11 sham and 12 UNX animals at 12 weeks. The values represent the mean ± SD; the *p*-values were obtained using unpaired Students’ *t*-test. The circles in box-plot distribution represent outliers.

**Figure 3:**
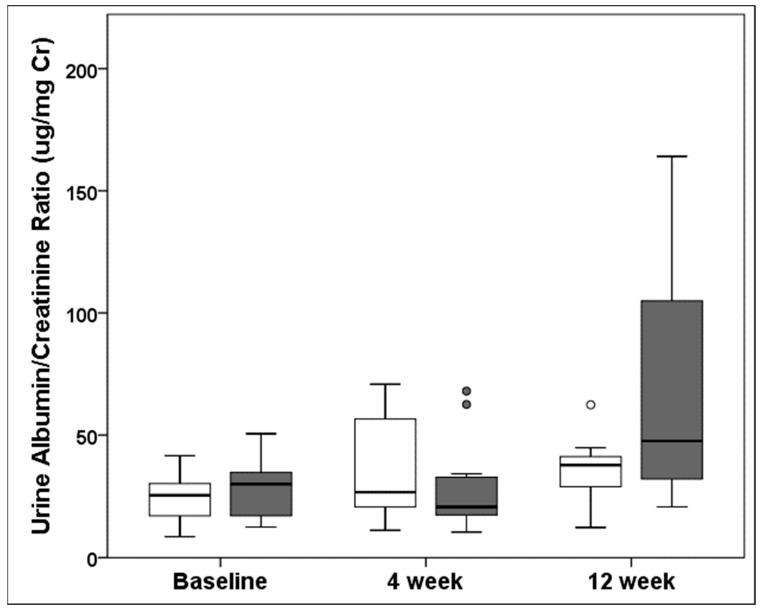
Box-plot distribution of urine albumin/creatinine ratios in TOPGAL animals at the baseline prior to unilateral nephrectomy (UNX) at 4 weeks of age in the sham and UNX groups and at 4 and 12 weeks. These measurements were performed on 12 sham and 12 UNX animals at 4 weeks following UNX and 11 sham and 12 UNX animals at 12 weeks following UNX. The baseline measurements of 4 weeks and 12 weeks animals were combined into a single baseline group. The values for the sham-treated mice are shown in the white boxplots, and those for the UNX mice are shown in the gray boxplots. The circles in box-plot distribution represent outliers.

(a) Body weight following unilateral nephrectomy. There was no significant difference in body weight at the baseline between the sham (*n* = 23, 20.1 ± 1.5 g) and the UNX (*n* = 24, 20.1 ± 1.8 g, *p* = 0.97) at the time of UNX or sham treatment at 4 weeks of age. Weight gain was lower at 4 weeks following UNX (mean difference −3.2 g, *p* = 0.002) but not at 12 weeks (mean difference +2.4 g, *p* = 0.32). Kidney weight could not be obtained due to the special perfusion protocol followed in these animals for X-gal staining at the time of euthanasia. Unilateral nephrectomy did impact growth at the early time point of 4 weeks, compared to the sham controls, but the UNX mice caught up with the sham-treated group by 12 weeks.
(b) Unilateral nephrectomy resulted in glomerular hypertrophy and increased the mesangial matrix. Quantitative morphometry was performed on the first 15 glomeruli, including 10 glomeruli from the outer cortical and 5 from the inner juxtamedullary regions, as the section was observed starting from one edge of the slide. Each glomerulus was analyzed for the glomerular area, glomerular perimeter, mesangial area, and mesangial cell count, which are shown in Table 1A–D and Figure 2A–E. A significant increase in the glomerular area over time was observed. The mean glomerular area increased by 11.6% and 15.1% in UNX, compared to the sham-treated animals, at 4 weeks and 12 weeks, respectively. The increase in the mean glomerular area was more marked in the juxtamedullary glomeruli, compared to the outer cortical glomeruli (17.1% vs. 8.6%), at 4 weeks but less so at 12 weeks (8.9% vs. 20.0%). Similarly, the mean glomerular perimeter increased by 5.4% and 8.6% in UNX, compared to the sham group, at 4 weeks and 12 weeks, respectively. The increase in the mean glomerular perimeter was more marked in the juxtamedullary glomeruli versus the outer cortical glomeruli (9.3% vs. 3.6%) at 4 weeks but less so at 12 weeks (5.4% vs. 10.7%). The mean mesangial area increased by 9.9% and 28.6% in UNX over the sham-treated animals at 4 weeks and 12 weeks, respectively. The increase in the mean mesangial area was exclusively in the juxtamedullary glomeruli, compared to the outer cortical glomeruli (31.5% vs. 0.4%), at 4 weeks but less so at 12 weeks (26.1% vs. 30.1%). In contrast to the mesangial matrix expansion, no significant increase in the mesangial cell count was observed in the glomeruli, except in the outer cortical glomeruli at 12 weeks. Together, these results show that unilateral nephrectomy in TOPGAL mice resulted in increased glomerular hypertrophy and increased the mesangial matrix, especially in the juxtamedullary glomeruli early and in the outer cortical glomeruli later, as part of the glomerular hemodynamic adaptation following UNX.
(c) Urine albumin excretion was increased in TOPGAL mice following unilateral nephrectomy. There was no difference at the baseline between the sham (24.2 ± 9.3 µg/mg Cr) and UNX TOPGAL mice (28.3 ± 11.3 µg/mg Cr, *p* = 0.189) at the time of surgery. At 4 weeks following unilateral nephrectomy, the urine albumin/creatinine ratios in the sham (*n* = 12, 36.1 ± 21.1 µg/mg Cr) and UNX (*n* = 12, 28.1 ± 18.7 µg/mg Cr, *p* = 0.338) animals were not different. However, the urine albumin/creatinine ratio increased significantly by 12 weeks in the UNX (*n* = 12, 66.3 ± 46.4 µg/mg Cr, *p* = 0.046), compared to the sham-treated group (*n* = 11, 35.3 ± 13.7 µg/mg Cr) of TOPGAL mice. The boxplot distribution of urine albumin/creatinine ratio at the baseline and following unilateral nephrectomy is shown in Figure 3.
(d) Unilateral nephrectomy resulted in the nuclear translocation of β-catenin in TOPGAL mice. The β-galactosidase expression in the reporter mouse (TOPGAL) was consistent with a nuclear translocation of β-catenin. The β-galactosidase expression was assessed by two different techniques, namely, immunofluorescence (*n* = 3 in each group) and X-gal staining (*n* = 3 in each group). Each animal was tested for β-galactosidase expression with only one method.

**Figure 4:**
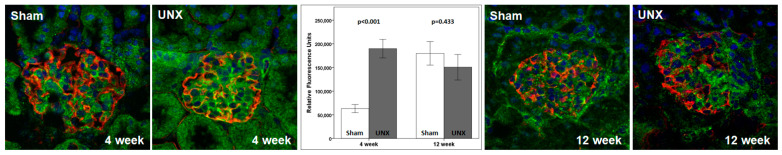
Confocal microscopy images show an increased expression of β-galactosidase (green fluorescence) as the relative fluorescence units/area at 4 weeks but not at 12 weeks (Table 2). Podocalyxin (red fluorescence) identifies the podocytes within the glomerulus. Unilateral nephrectomy is associated with glomerular hypertrophy. The bar graph shows the net relative fluorescence units/glomerulus.

**Figure 5:**
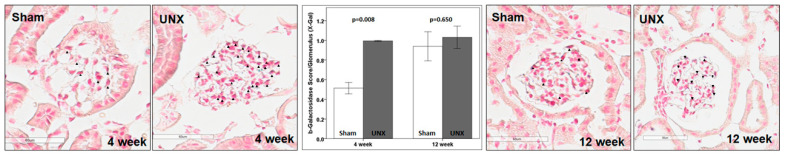
X-gal staining for β-galactosidase in TOPGAL animals at 4 and 12 weeks following unilateral nephrectomy or sham treatment at 4 weeks of age. The X-gal staining was measured using a semi-quantitative score of 0–4 of all the glomeruli in the section. The X-gal staining showed an increased expression of β-galactosidase at 4 weeks but not at 12 weeks. The arrowheads mark the X-gal positive podocytes. The bar graph shows the mean ± SD of the β-galactosidase expression at 4 and 12 weeks following UNX.

Confocal microscopy showed that the relative fluorescence units/area of the glomerulus was significantly increased (*p* < 0.001) at 4 weeks following UNX (Table 2). Since unilateral nephrectomy is associated with glomerular hypertrophy, we also assessed the net fluorescence/glomerulus (Figure 4). The increase in β-galactosidase expression at 4 weeks following UNX was further supported by X-gal staining (*p* = 0.008), which was measured by a semi-quantitative score of 0–4 across all the glomeruli in the section (Table 2 and Figure 5). However, the increased β-galactosidase expression dissipated by 12 weeks following UNX. The mean β-galactosidase expression was not different in UNX animals, compared to the sham-treated group, at 12 weeks (Table 2 and Figure 4 and Figure 5). 

A consistent increase in the β-galactosidase expression at 4 weeks following UNX using two different methods suggests a definite increase in the activation and nuclear translocation of the transcription factor, β-catenin. As shown below, the results also show that β-catenin was phosphorylated at Ser552 in podocytes. The observed increase in the reporter β-galactosidase expression in the early period following UNX indicates the significance of β-catenin in the adaptive response to the loss of one kidney.

**Figure 6:**
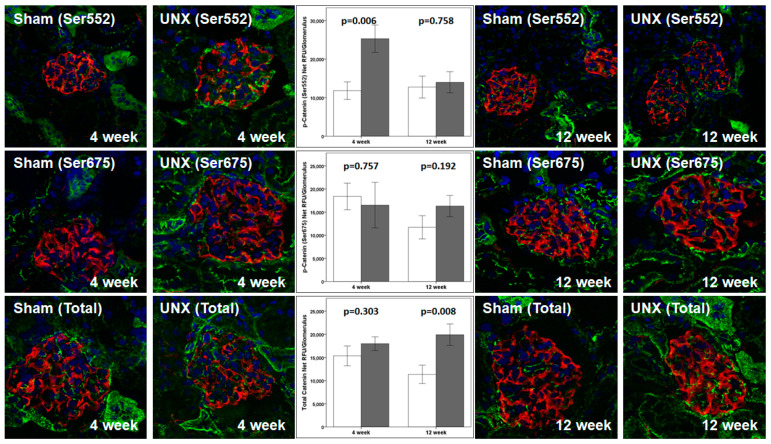
Confocal microscopy images show the expression of β-catenin (green fluorescence) and Podocalyxin (red fluorescence) in TOPGAL animals at 4 and 12 weeks following unilateral nephrectomy or sham treatment at 4 weeks of age. Unilateral nephrectomy is associated with glomerular hypertrophy. The bar graph shows the net relative fluorescence units/glomerulus. The values are shown as the mean relative fluorescence units [RFU] ± SD; the *p*-values were obtained using unpaired Students’ *t*-test.

(e) Unilateral nephrectomy resulted in increased levels of β-catenin. Confocal microscopy showed a significant increase in the phospho-β-catenin (Ser552) relative fluorescence units/area of the glomerular at 4 weeks (*p* = 0.005) but not at 12 weeks (*p* = 0.935) following UNX (Table 3). No changes were detected in the phospho-β-catenin (Ser675) relative fluorescence units/area of the glomerulus at 4 weeks (*p* = 0.170) or at 12 weeks (*p* = 0.197) following UNX (Table 3). Interestingly, confocal microscopy for RFU/area of the glomerulus also showed a significant increase in the total catenin expression (*p* = 0.027) at 12 weeks but not at 4 weeks (*p* = 0.111) following UNX (Table 3). Unilateral nephrectomy is associated with glomerular hypertrophy, and we therefore also assessed the net fluorescence/glomerulus for phospho-β-catenin (Ser552), phospho-β-catenin (Ser675), and Total β-catenin (Figure 6). The results showed an increased phospho-β-catenin (Ser552) at 4 weeks (*p* = 0.006) and increased the Total β-catenin at 12 weeks (*p* = 0.008), while there was no change in phospho-β-catenin (Ser675). Thus, there was an increase in phospho-β-catenin (Ser552) in the kidneys following unilateral nephrectomy at 4 weeks and Total- β-catenin at 12 weeks, but not for phospho-β-catenin (Ser675).

### 3.2. Fluid Flow Shear Stress on Podocytes Resulted in a Sustained Increase in the Nuclear Translocation of Phospho-β-Catenin (Ser552)

**Figure 7:**
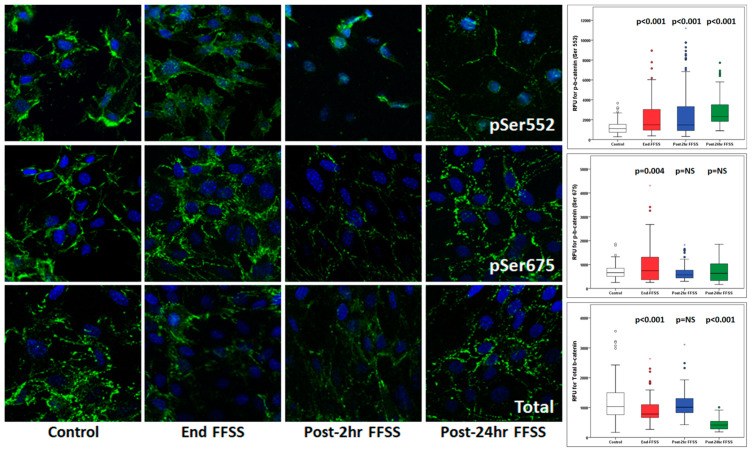
Phosphorylated (Ser552 and Ser675) and non-phosphorylated (Total) β-catenin in the nuclei of podocytes exposed to fluid flow shear stress (FFSS). Untreated (Control) cells were compared with podocytes exposed to FFSS at 2 dynes/cm^2^ for 2 h, which were observed at the end of FFSS (End-FFSS), Post-2 h FFSS, and Post-24 h FFSS. Representative immunofluorescence images are shown. The box-plot distribution for the relative fluorescence units (RFU) in the nuclei of podocytes is on the far right.

Western blot analysis of cell lysates from cultured podocytes exposed to FFSS showed an increased expression of phospho-β-catenin (Ser552) but not phospho-β-catenin (Ser675) [14]. In this study, we performed confocal imaging to localize and quantitate the Total (non-phosphorylated) and phosphorylated β-catenin in the nucleus. We measured the total fluorescence in the nucleus (in relative fluorescence units, RFU) in at least 50 nuclei from cells in 3–5 images following FFSS on podocytes (*n* = 3). At the end of the FFSS treatment, there was a significant increase in phosphorylated phospho-β-catenin (Ser552) and phospho-β-catenin (Ser675) and a significant decrease in the Total (non-phosphorylated) β-catenin in the nucleus (Table 4 and Figure 7). Phospho-β-catenin (Ser675) returned to the baseline by Post-2 h, which is in contrast to phospho-β-catenin (Ser552), as the latter continued to increase over time. The total (non-phosphorylated) β-catenin at Post-24 h FFSS was lower than the baseline. These results show that phospho-β-catenin (Ser552) and not phospho-β-catenin (Ser675) plays a key role in response to FFSS in podocytes, as demonstrated here by confocal imaging studies and earlier by Western blotting [14].

### 3.3. Analysis of the Gene Expression Omnibus Dataset on Unilateral Nephrectomy Corroborated In Vivo and In Vitro Studies

Bioinformatic analysis of GSE53996 identified 1722 microarray probes corresponding to 898 mapped genes that were differentially expressed (*p* < 0.05) in the solitary kidney after unilateral nephrectomy in mice. To investigate the molecular mechanisms underlying unilateral nephrectomy, the microarray data were submitted for IPA core analysis. IPA core analysis predicted the canonical pathways and upstream regulators affected by unilateral nephrectomy. The top enriched categories of canonical pathways are listed in Table 5. In alignment with our previous work using FFSS-treated podocytes, the present bioinformatic analysis showed changes in the G-protein coupled receptor signaling, Wnt/β-catenin, actin cytoskeleton, and cAMP-mediated signaling [10,12,14,15]. Upstream regulator analysis predicted the G-protein coupled receptors (Table 6), transcription factors (Table 7), and kinases (Table 8) associated with the observed differential expression. Thus, as with our previous findings, the current bioinformatic analysis also identified the EP2 (PTGER2) receptor as the G-protein coupled receptor, AKT and GSK3β as kinases, and β-catenin as an upstream transcription factor. These findings through bioinformatics analyses of a publicly available dataset generated by an independent group further provides evidence of the relevance of G-coupled protein signaling through the prostanoid receptor, EP2, in the “PGE2-COX2-EP2” axis and the role of β-catenin in the “AKT-GSK3β-β-catenin” pathway downstream of EP2 activation in podocytes previously reported by us in in vitro studies. These results advance our understanding of the interactions between these molecules, which will be valuable in future work.

As part of our ongoing work to understand the role of FFSS-mediated injury in solitary kidney, we previously demonstrated that FFSS activated the “PGE_2_-COX2-EP2” axis and upregulated the “AKT-GSK3β-β-catenin” pathway in podocytes in vitro and validated the role of a COX2 and EP2 in vivo model of solitary kidney [10,11,12,14,15]. In the current study, we now show the importance of the transcription factor, β-catenin, in vivo following unilateral nephrectomy in a reporter TOPGAL mouse and, additionally, confirm the role of phospho-β-catenin (Ser552) in response to FFSS in a solitary kidney model both in vivo and in vitro. The results from our previous and current study are further supported by bioinformatic analyses of an independent dataset available in the public domain. 

## 4. Discussion

Hyperfiltration is a major underlying factor in the onset and progression of CKD, which is associated with podocyte injury, resulting in impairment of the glomerular filtration barrier and a decreased renal function over time. The present in vivo results validate previous in vitro studies that described the significance of β-catenin signaling in FFSS-treated podocytes [14,15]. The present results show glomerular hypertrophy and an increased mesangial matrix in unilaterally nephrectomized TOPGAL mice. The glomerular hypertrophy was more marked in juxtaglomerular glomeruli in the early period (4 weeks) and in cortical glomeruli in the later period (12 weeks). The results also show an increased albuminuria at 3 months, increased nuclear translocation of β-catenin at 4 weeks, phosphorylation of β-catenin at Ser552 and not Ser675 in UNX mice, which was further complemented by in vitro studies in podocytes, and the bioinformatic analysis of publicly available independent datasets supports our previously reported activation of “COX2-PGE_2_-EP2” and “AKT-GSK3β-β-catenin” in podocytes in vitro. These studies advance our understanding of the mechanism of CKD progression to ESRD in adult kidney donors with acquired solitary kidney and in children born with solitary functioning kidney. 

Glomerular hypertrophy is considered a hallmark of decreasing kidney function and proteinuric disease. Key in vivo findings, glomerular hypertrophy, and an increased mesangial matrix in TOPGAL mice were evident by 4 weeks post-UNX and showed further progression at 12 weeks (Figure 2 and Table 1). Previously, we reported an increased glomerular diameter and area as early as 2–3 weeks in C57BL/6J mice and by 4 weeks in sv129 mice following UNX [9,10]. Celsi et al. [1,2] reported an increase in glomerular diameter and area at ~7 weeks and not at ~2 weeks following UNX in Sprague Dawley rats. To further refine and advance our findings, we examined and compared the adaptive changes in cortical and juxtamedullary glomeruli. The glomerular hypertrophy was more marked in the juxtamedullary region at 4 weeks and in the cortical region by 12 weeks. A centrifugal pattern of renal maturation from the juxtamedullary region towards the superficial cortical layers leads to subtle regional differences in the structure and function of the nephrons [31]. Thus, the larger juxtamedullary glomeruli, compared to the cortical glomeruli, in the present study correspond with normal size differences (Table 1). Vascular damage from hypertension occurs first and more severely in the juxtamedullary glomeruli [32]. A pathologic glomerular lesion of focal segmental glomerulosclerosis is most common in or restricted to the juxtamedullary cortex [33]. Thus, the presently observed early glomerular adaptive changes in the juxtamedullary region are of special significance in the development of secondary focal segmental glomerulosclerosis from hyperfiltration-mediated injury. 

The unilaterally nephrectomized TOPGAL mice showed an increased urinary albumin by 12 weeks, which was not evident at 4 weeks (Figure 3). Recently, we showed sv129 mice with increased urine albumin following unilateral nephrectomy [10]. The onset of albuminuria is preceded by increased albumin in the glomerular filtrate, as a result of a damaged glomerular filtration barrier. Using an in vitro assay developed in our laboratory, we demonstrated that a variety of agents and conditions increase glomerular albumin permeability (P_alb_). We have also demonstrated that an increase in P_alb_ precedes the onset of proteinuria in animal models of diabetes, hypertension, radiation nephropathy, puromycin-induced nephrosis, and focal segmental glomerulosclerosis [34,35,36,37]. We have also demonstrated that treatment of isolated rat glomeruli with PGE_2_ or FFSS increased P_alb_, which could be blocked by indomethacin, indicating a role of PGE_2_ [10,38,39]. Thus, previously reported work and present observations show that UNX-induced increases in FFSS damage the glomerular filtration barrier causing increased albumin permeability and, subsequently, proteinuria. Similarly, children born with solitary kidney start to develop hypertension and proteinuria in late adolescence, suggesting that glomerular injury due to hyperfiltration gradually leads to proteinuria over several years in these children [40,41]. Thus, morphological changes, glomerular hypertrophy, and an increased mesangial matrix complement the observed functional outcome in TOPGAL mice following UNX. 

The TOPGAL mouse is a specific reporter strain with β-galactosidase activity, as a direct in vivo readout for activated β-catenin signaling. Using the TOPGAL mice, we demonstrated an increase in the β-galactosidase activity, as direct evidence for the in vivo activation of β-catenin following UNX. We used two different methods (described under Methods) to evaluate the β-galactosidase expression in TOPGAL and the data from each method support this finding. The increased β-galactosidase activity (i.e., β-catenin signaling) was detectable at 4 weeks following UNX and returned to the baseline by 12 weeks. We anticipated a transient activation of β-catenin signaling, since it is a transcription factor, and its activation would cause progressive changes. 

A small but significant magnitude of increase in β-galactosidase activity at 4 weeks but not at 12 weeks in β-galactosidase expression is consistent with other reports. For example, the β-galactosidase activity in TOPGAL mice is observed during fetal and neonatal periods in the skeleton and is not detectable in mature animals, unless induced by mechanical loading or fracture in vivo [42,43]. In vivo bone loading in 5- and 12-months-old TOPGAL mice for 1 day and 5 day was shown to result in a β-galactosidase expression in 5% to 10% of osteocytes that peaked at 24 h and declined to baseline by 72 h [44,45]. Additionally, the expression of β-galactosidase in callus and periosteum following a fracture stimulus was observed in TOPGAL mice. Treatment with GSK-3β inhibitor resulted in an increased β-catenin activation and rapid fracture healing, showing the importance of β-catenin in response to injury [43]. Thus, stress or injury stimulate β-catenin signaling in a TOPGAL mouse model of UNX, which is similar to that observed in models of bone loading and fracture. 

β-catenin phosphorylation by casein kinase (Ser45) followed by GSK3β (Ser33,37 and Thr41) directs it toward degradation through the ubiquitin-proteasome degradation pathway [18]. In contrast, phosphorylation by AKT (predominantly) at Ser552 and by PKA (predominantly) at Ser675 results in the nuclear translocation of β-catenin for transcriptional activity via LEF/TCF [16,17]. We previously identified β-catenin, a transcription factor, in podocytes treated with FFSS through Enrichr and IPA bioinformatic analyses as an upstream regulator [14]. Western blot analysis of podocytes treated with PGE_2_ (1 µM) or FFSS showed an increased phosphorylation of β-catenin (Ser552) but not β-catenin (Ser675) [14]. Western blotting also showed the upregulation of CD44/ERBB2/β-catenin pathway in podocytes following FFSS [15]. 

Presently, we evaluated the localization and movement of phospho-β-catenin (Ser552), phospho-β-catenin (Ser675), and total β-catenin using confocal microscopy. The results (Table 4 and Figure 7) show the translocation to and accumulation of phospho-β-catenin (Ser552) in the nucleus. A transient increase in phospho-β-catenin (Ser675) suggests a role of AKT-GSK3β-β-catenin, compared to cAMP-PKA-β-catenin, as observed previously [14]. Additionally, confocal microscopy of the kidney from unilaterally nephrectomized TOPGAL mice showed an upregulated phospho-β-catenin (Ser552) at 4 weeks and Total β-catenin at 12 weeks, while phospho-β-catenin (Ser675) remained unchanged (Table 3 and Figure 6). The significance of β-catenin signaling is also highlighted by other reports, as it was found to mediate oxidative stress-induced podocyte dysfunction in vitro and in vivo [19]. Podocyte injury and albuminuria caused by Adriamycin were ameliorated in β-catenin knock-out mice, and the over-expression of podocyte β-catenin was also detected in human diabetic nephropathy and focal segmental glomerulosclerosis [20]. Additionally, treatment of osteocytes with PGE_2_ results in the EP2-activated phosphorylation of GSK3β and increased nuclear translocation of β-catenin, followed by binding with LEF/TCF and transcriptional regulation of genes, including COX2, suggesting a role of β-catenin in skeletal physiology [46,47,48,49,50]. Thus, understanding the importance of β-catenin in FFSS-mediated injury in solitary kidney will be critical for developing strategies to treat the progression of CKD in transplant donors with acquired solitary kidney and children born with solitary functioning kidneys.

Considering its potential clinical significance, we adopted a third pre-clinical approach to corroborate our finding that hyperfiltration-induced kidney injury causes the upregulation of β-catenin signaling. To this end, we performed bioinformatic analysis using publicly available dataset in the Gene Expression Omnibus. As shown in Table 5, Table 6, Table 7 and Table 8, the IPA analyses showed G-protein coupled receptor signaling as the main pathway, with prostanoid receptor EP2 as the G-protein-coupled protein receptor, AKT1, GSK3β, Erbb, and mTOR as the kinases, and β-catenin as the transcription factor. Previously, we validated the phosphorylation of AKT1 (Ser473), GSK3β (Ser9), and β-catenin (Ser552 and Ser675) [14]. Additionally, we reported the altered phosphorylation of Erbb2 (Tyr1221/1222), mTOR (Ser2448), ERK1/2 (Thr202/Tyr204), and p38MAPK (Thr180/Tyr182), but not PKA (Thr197) [14,15]. Thus, the mechanoperception and mechanotransduction pathways that we have previously identified were validated from a dataset generated by other investigators [29,30]. Therefore, based on our previous work, the present in vitro and in vivo results, and an analysis of a publicly available dataset, we propose β-catenin signaling to be relevant for understanding hyperfiltration-induced injury in solitary kidney. 

Since PGE_2_ receptors are known to activate diverse signaling pathways, the activity of several proteins is likely affected due to FFSS. Of the four PGE_2_ receptors, only EP2 activation results in the nuclear translocation of activated β-catenin through five known intermediate mechanisms (Figure 1). EP2 is a G-protein-coupled receptor that activates the heterotrimeric (αβγ) Gs protein, leading to (i) the release of the Gβγ complex, activating PI3K/AKT, which phosphorylates and inactivates GSK3β [51,52], (ii) binding of the Gα complex to Axin and the release of β-catenin from the Axin-β-catenin-GSK3β complex, (iii) generation of cAMP and activation of PKA, which activate β-catenin [53,54,55], (iv) recruitment of β-arrestin-1 and phosphorylation of Src-kinase, which transactivates the EGFR signaling network of the PI3K/AKT, HGF/c-Met, and Ras/ERK pathways [56,57,58], and (v) activation of PI3K/AKT leading to the activation of ERK [59]. Studies suggest that the phosphorylation of β-catenin at Ser552 or Ser675 induces β-catenin translocation to the nucleus, with AKT preferentially phosphorylating β-catenin at Ser552, while PKA preferentially phosphorylating it at Ser675 [16,17,55,60]. Thus, there is a strong link between G-protein-coupled receptor signaling via the EP2 receptor and β-catenin signaling, which could be targeted to mitigate injury. 

We believe that there are at least two clinical scenarios where susceptibility to CKD needs special attention, as the reason(s) for the progression to ESRD is still unclear, and specific treatment(s) are not available: (a) in adults following kidney donation and (b) in children born with solitary functioning kidney. We recently reviewed the accumulating evidence that living kidney donation increases the risk of ESRD in donors, despite stringent screening [61]. The risk of ESRD in donors is 8.3-fold higher, compared to eligible healthy individuals, as reported in a meta-analysis of 52 studies [62,63,64,65]. There is a cumulative increase in the incidence of ESRD over time following kidney donation, with a reported mean interval of 27.1 ± 9.8 years [66,67,68]. Secondly, a considerable number of children born with solitary kidney develop albuminuria during adolescence and progress to end-stage renal disease as young adults [69,70,71,72]. Children with SFK manifest renal injury at a median age of ~15 years with hypertension, proteinuria, and/or an eGFR of less than 60 mL/min/1.73 m^2^ [69,70,71,72]. The significance of FFSS in hyperfiltration-mediated glomerular injury in the progression of CKD is being better understood and appreciated. With growing evidence, we propose EP2 and/or β-catenin as suitable targets for mitigating glomerular injury.

## Figures and Tables

**Table 1:** (**A**–**D**): The table shows the glomerular area (**A**), glomerular perimeter (**B**), mesangial area (**C**), and mesangial cell count (**D**) in TOPGAL animals at 4 and 12 weeks following unilateral nephrectomy or sham treatment at 4 weeks of age. The measurements were performed on 15 glomeruli (10 outer cortical glomeruli and 5 inner juxtamedullary glomeruli) in a kidney section stained with Periodic Acid Schiff stain. This was performed on 12 sham and 12 UNX animals at 4 weeks and 11 sham and 12 UNX animals at 12 weeks. The values are shown as the Mean ± SD; the p-values were obtained using unpaired Students’ *t*-test.

**A. Glomerular Area (µm^2^)**		**4 Weeks**	***p*-Value**	**12 Weeks**	***p*-Value**
All Glomeruli (*n* = 15)	Sham	4039 ± 1494		4352 ± 1700	
	UNX	4507 ± 1719	0.006	5010 ± 1741	<0.001
Outer Cortical Glomeruli (*n* = 10)	Sham	3711 ± 1165		3840 ± 1119	
	UNX	4031 ± 1210	0.037	4609 ± 1230	<0.001
Juxtamedullary Glomeruli (*n* = 5)	Sham	4679 ± 1833		5349 ± 2152	
	UNX	5480 ± 2156	0.031	5825 ± 2277	0.251
**B. Glomerular Perimeter (µm)**		**4 Weeks**	***p*-Value**	**12 Weeks**	***p*-Value**
All Glomeruli (*n* = 15)	Sham	247.9 ± 46.7		252.3 ± 46.9	
	UNX	261.3 ± 50.2	0.009	274.0 ± 46.8	<0.001
Outer Cortical Glomeruli (*n* = 10)	Sham	239.9 ± 38.3		239.0 ± 34.7	
	UNX	248.5 ± 40.0	0.091	264.6 ± 36.4	<0.001
Juxtamedullary Glomeruli (*n* = 5)	Sham	263.3 ± 57.1		278.2 ± 56.2	
	UNX	287.7 ± 58.6	0.023	293.3 ± 58.5	0.161
**C. Mesangial Area (µm^2^)**		**4 Weeks**	***p*-Value**	**12 Weeks**	***p*-Value**
All Glomeruli (*n* = 15)	Sham	970 ± 444		1061 ± 526	
	UNX	1066 ± 608	0.089	1364 ± 670	<0.001
Outer Cortical Glomeruli (*n* = 10)	Sham	1012 ± 432		1014 ± 499	
	UNX	1016 ± 529	0.950	1319 ± 616	<0.001
Juxtamedullary Glomeruli (*n* = 5)	Sham	889 ± 458		1151 ± 567	
	UNX	1169 ± 737	0.014	1456 ± 766	0.016
**D. Mesangial Cell Count (*n*)**		**4 Weeks**	***p*-Value**	**12 Weeks**	***p*-Value**
All Glomeruli (*n* = 15)	Sham	17.1 ± 4.7		17.2 ± 6.1	
	UNX	17.8 ± 5.8	0.191	19.1 ± 5.8	0.003
Outer Cortical Glomeruli (*n* = 10)	Sham	17.3 ± 5.0		17.0 ± 5.7	
	UNX	17.9 ± 5.6	0.452	19.0 ± 5.5	0.006
Juxtamedullary Glomeruli (*n* = 5)	Sham	16.4 ± 4.2		17.7 ± 6.9	
	UNX	17.6 ± 6.1	0.236	19.3 ± 6.2	0.196

**Table 2:** Expression of β-galactosidase determined using immunofluorescence and X-gal staining in TOPGAL animals at 4 and 12 weeks following unilateral nephrectomy or sham treatment at 4 weeks of age. The measurements were performed in 3 sham and 3 UNX animals at 4 weeks and 12 weeks in separate sets of animals. No single animal was studied using two methods to demonstrate a change in β-galactosidase. The values are shown as the mean ± SD; the *p*-values were obtained using an unpaired Students’ *t*-test. RFU- Relative Fluorescence Units.

β-Galactosidase	Group	4 Weeks	*p*-Value	12 Weeks	*p*-Value
Immunofluorescence (RFU/µm^2^)	Sham	14.8 ± 8.5		48.5 ± 29.5	
*n* = 3	UNX	37.1 ± 9.8	<0.001	36.0 ± 24.4	0.16
X-Gal Score (0–4)	Sham	0.51 ± 0.10		0.94 ± 0.26	
*n* = 3	UNX	0.99 ± 0.08	0.008	1.03 ± 0.20	0.650

**Table 3:** Expression of β-catenin using immunofluorescence in TOPGAL animals at 4 and 12 weeks following unilateral nephrectomy or sham treatment at 4 weeks of age. Fluorescence measurements were performed in 3 Sham and 3 UNX animals at 4 weeks and 12 weeks. The values are shown as the mean relative fluorescence units [RFU] ± SD; the p-values were obtained using unpaired Students’ *t*-test.

β-Catenin in TOPGAL (RFU/µm^2^)	Group	4 Weeks	*p*-Value	12 Weeks	*p*-Value
p-β-catenin (Ser552)	Sham	3.10 ± 1.28		2.72 ± 1.78	
	UNX	5.85 ± 2.25	0.005	2.79 ± 1.56	0.935
p-β-catenin (Ser675)	Sham	3.69 ± 1.32		2.20 ± 1.18	
	UNX	3.07 ± 0.79	0.170	2.93 ± 1.17	0.197
Total β-catenin	Sham	3.41 ± 1.86		2.65 ± 1.65	
	UNX	4.26 ± 1.86	0.111	4.16 ± 2.15	0.027

**Table 4:** Levels of phosphorylated and non-phosphorylated (Total) transcription factor β-catenin in the nuclei of podocytes exposed to fluid flow shear stress (FFSS) using immunofluorescence microscopy. The fluorescence from untreated (Control) cells was compared with podocytes exposed to FFSS at 2 dynes/cm^2^ for 2 h at the end of FFSS, (End-FFSS), Post-2 h FFSS, and Post-24 h FFSS. The relative fluorescence units (RFU) values are shown as the Mean ± SD; the p-values were obtained using Univariate Analysis of Variance with pairwise comparison, corrected using the Bonferroni post-hoc test.

Nuclear Fluorescence (RFU)	Control	End-FFSS	Post-2 h FFSS	Post-24 h FFSS
phospho-β-catenin (Ser552)	1246.5 ± 717.6	2175.7 ± 1677.2	2584.9 ± 2529.0	2838.3 ± 1554.5
Control vs. (*p* value)	-	<0.001	<0.001	<0.001
End-FFSS vs. (*p* value)	-	-	0.230	0.040
Post-24 h FFSS vs. (*p* value)	-	-	-	1.000
phospho-β-catenin (Ser675)	725.8 ± 321.0	965.2 ± 839.3	678.7 ± 339.3	700.7 ± 418.1
Control vs. (*p* value)	-	0.004	1.000	1.000
End-FFSS vs. (*p* value)	-	-	0.002	0.002
Post-24 h FFSS vs. (*p* value)	-	-	-	1.000
β-catenin (Total)	1177.6 ± 621.1	919.6 ± 411.9	1100.1 ± 399.1	443.6 ± 200.9
Control vs. (*p* value)	-	<0.001	0.922	<0.001
End-FFSS vs. (*p* value)	-	-	0.013	<0.001
Post-24 h FFSS vs. (*p* value)	-	-	-	<0.001

**Table 5:** The top 25 canonical pathways identified by Ingenuity Pathway Analysis of the Gene Expression Omnibus dataset on unilaterally nephrectomized mice (GSE 53996).

	Ingenuity Canonical Pathways	−log(*p*-Value)
1	G-Protein Coupled Receptor Signaling	6.16
2	Role of Osteoblasts, Osteoclasts and Chondrocytes in Rheumatoid Arthritis	4.82
3	cAMP-mediated signaling	4.71
4	CREB Signaling in Neurons	4.68
5	Axonal Guidance Signaling	4.65
6	Human Embryonic Stem Cell Pluripotency	4.37
7	Wnt/β-catenin Signaling	4.22
8	Synaptic Long-Term Depression	4.21
9	Actin Cytoskeleton Signaling	4.14
10	Ephrin Receptor Signaling	4.14
11	EIF2 Signaling	3.91
12	Renal Cell Carcinoma Signaling	3.9
13	Protein Ubiquitination Pathway	3.85
14	Gαi Signaling	3.8
15	Androgen Signaling	3.66
16	Molecular Mechanisms of Cancer	3.66
17	Regulation of the Epithelial-Mesenchymal Transition Pathway	3.63
18	Role of Oct4 in Mammalian Embryonic Stem Cell Pluripotency	3.6
19	Role of Macrophages, Fibroblasts and Endothelial Cells in Rheumatoid Arthritis	3.43
20	CCR3 Signaling in Eosinophils	3.39
21	Hepatic Fibrosis/Hepatic Stellate Cell Activation	3.39
22	Glioblastoma Multiforme Signaling	3.26
23	Ephrin B Signaling	3.24
24	Natural Killer Cell Signaling	3.21
25	Methylglyoxal Degradation III	3.19

**Table 6:** The G-coupled protein receptors identified as Upstream Regulators by Ingenuity Pathway Analysis of the Gene Expression Omnibus dataset on unilaterally nephrectomized mice (GSE 53996).

Upstream Regulator	Molecule Type	Activation z-Score	*p*-Value of Overlap
PTGER2	g-protein coupled receptor	1.49	0.00954
CXCR4	g-protein coupled receptor	0.822	0.000305
SMO	g-protein coupled receptor	0.762	0.00921
CNR1	g-protein coupled receptor	−0.042	0.006
CXCR2	g-protein coupled receptor		0.0247
ACKR3	g-protein coupled receptor		0.0268
LHCGR	g-protein coupled receptor		0.0301

**Table 7:** The transcription factors identified as Upstream Regulators by Ingenuity Pathway Analysis of the Gene Expression Omnibus dataset on unilaterally nephrectomized mice (GSE 53996). The top 50 of the 230 transcription factors identified by the Ingenuity Pathway Analysis.

	Upstream Regulator	Expr Log Ratio	Predicted Activation State	Activation z-Score	*p*-Value of Overlap
1	CTNNB1	0.552	Activated	2.615	1.13 × 10^10^
2	HTT			−0.396	1.72 × 10^9^
3	NFE2L2		Activated	3.387	9.76 × 10^9^
4	TP53			1.482	0.000000011
5	SP1		Activated	2.967	9.35 × 10^8^
6	HDAC1	0.655		0.221	0.000000394
7	FOS			1.94	0.00000153
8	SMARCA4		Activated	3.718	0.00000389
9	GATA6			−1.178	0.00000608
10	FOXA1	0.651		0.712	0.00000686
11	KLF4			1.72	0.00000717
12	BRCA1			1.418	0.00000737
13	TCF7L2		Activated	3.079	0.000011
14	SOX2			1.166	0.0000111
15	RUNX1		Activated	2.153	0.0000141
16	MYC		Activated	2.354	0.0000185
17	PTF1A			−0.044	0.0000199
18	HIF1A		Activated	2.22	0.0000202
19	GLI1		Activated	2.486	0.0000259
20	EZH2			−0.374	0.0000296
21	HNF4A			0.72	0.000032
22	CEBPA			1.81	0.0000429
23	GBX2				0.0000443
24	SOX10			0.798	0.0000493
25	EP300	0.537		1.248	0.0000554
26	E2F1			1.08	0.000056
27	CREBBP			1.08	0.000074
28	NFE2L1			0.896	0.0000816
29	LHX5	0.581			0.0000998
30	WT1			1.38	0.00011
31	PPARGC1A		Activated	3.064	0.000165
32	SIX1			1.195	0.000179
33	SMAD5			−0.15	0.000179
34	POU5F1	0.505		0.832	0.000186
35	GATA1	1.352		1.431	0.000188
36	SOX3			−0.302	0.000241
37	FOXC1			1.4	0.000294
38	NFKBIA			−0.019	0.000321
39	MSGN1		Activated	2	0.000482
40	EPAS1			1.945	0.000485
41	CDKN2A	0.869		−0.114	0.000577
42	ARNT2		Activated	2.429	0.000629
43	HNF1A			0.861	0.000886
44	SIM1		Activated	2.457	0.000912
45	TP63			1.457	0.000939
46	JUN		Activated	2.079	0.00104
47	SMAD4			0.442	0.00105
48	LHX1			0.17	0.00105
49	SP3	0.499	Activated	2.078	0.00109
50	MECP2				0.00115

**Table 8:** The kinases identified as Upstream Regulators by the Ingenuity Pathway Analysis of the Gene Expression Omnibus dataset on unilaterally nephrectomized mice (GSE 53996).

	Upstream Regulator	Expr Log Ratio	Predicted Activation State	Activation z-Score	*p*-Value of Overlap
1	EGFR			1.637	0.0000143
2	ERBB3	0.431		0	0.0000514
3	GSK3B			−0.772	0.000473
4	BMPR1B			0.555	0.00102
5	TGFBR2			0.36	0.00109
6	ERBB2			1.424	0.00148
7	MUSK	0.413			0.0018
8	TNK2				0.00215
9	CDK5			1.188	0.00225
10	FGFR2	−0.337		−0.413	0.0027
11	ZAP70			1.463	0.00412
12	INSR		Activated	2.959	0.0044
13	PRKAA2			−0.508	0.00469
14	MAPK9			−0.565	0.00512
15	MTOR			0.974	0.00652
16	CDK5R1				0.00677
17	DCLK1				0.00679
18	BMPR1A		Activated	2.394	0.00867
19	MAP4K4		Inhibited	−2.309	0.00995
20	KDR				0.011
21	CCND3			−1.154	0.0123
22	PIM1	0.746		1.331	0.0132
23	RET	−0.811		0.24	0.015
24	STK11			−0.277	0.0151
25	MAP2K3			1.342	0.0156
26	PRKAA1			−0.213	0.0156
27	MAP2K1			1.784	0.0158
28	ERBB4			−0.954	0.0182
29	PRKCA			1.969	0.0191
30	NDRG1			−0.374	0.0194
31	GRB2			0	0.0209
32	PIK3CA			0.2	0.0225
33	AKT1		Activated	3.144	0.0246
34	PLK4			1	0.0268
35	FLT3				0.0272
36	PTK6				0.0285
37	EPHB4	0.874		0.816	0.03
38	PLK2			1	0.0306
39	CHEK2				0.0355
40	IRAK2				0.0355
41	EFNB2				0.0355
42	PRKCI			0.577	0.039
43	IRAK3				0.0417
44	IKBKB			0.247	0.0422
45	TSSK3				0.0464
46	CAMK2N1				0.0464
47	AMHR2				0.0464
48	C8orf44-SGK3/SGK3				0.0464
49	WNK3				0.0464
50	MAP3K20				0.0464
51	STK35				0.0464
52	PRKCB			−0.391	0.048
53	RPS6KA5				0.0484

## Data Availability

GEO dataset, GSE 53996, “Effects of high-fat induced obesity on gene expression in mouse kidney”. GEO dataset samples: GSM1305179 LFD-sham_1, GSM1305180 LFD-sham_2, GSM1305181 LFD-sham_3, GSM1305182 LFD-sham_4, GSM1305183 LFD-UNX_1, GSM1305184 LFD-UNX_2, GSM1305185 LFD-UNX_3, and GSM1305186 LFD-UNX_4.
